# Sharp Second-Order Pointwise Asymptotics for Lossless Compression with Side Information

**DOI:** 10.3390/e22060705

**Published:** 2020-06-25

**Authors:** Lampros Gavalakis, Ioannis Kontoyiannis

**Affiliations:** Department of Engineering, University of Cambridge, Trumpington Street, Cambridge CB2 1PZ, UK; ik355@cam.ac.uk

**Keywords:** entropy, lossless data compression, side information, conditional entropy, central limit theorem, law of the iterated logarithm, conditional varentropy

## Abstract

The problem of determining the best achievable performance of arbitrary lossless compression algorithms is examined, when correlated side information is available at both the encoder and decoder. For arbitrary source-side information pairs, the conditional information density is shown to provide a sharp asymptotic lower bound for the description lengths achieved by an arbitrary sequence of compressors. This implies that for ergodic source-side information pairs, the conditional entropy rate is the best achievable asymptotic lower bound to the rate, not just in expectation but with probability one. Under appropriate mixing conditions, a central limit theorem and a law of the iterated logarithm are proved, describing the inevitable fluctuations of the second-order asymptotically best possible rate. An idealised version of Lempel-Ziv coding with side information is shown to be universally first- and second-order asymptotically optimal, under the same conditions. These results are in part based on a new almost-sure invariance principle for the conditional information density, which may be of independent interest.

## 1. Introduction

It is well-known that the presence of correlated side information can potentially offer dramatic benefits for data compression [1,2]. Important applications where such side information is naturally present include the compression of genomic data [3,4], file and software management [5,6], and image and video compression [7,8].

In practice, the most common approach to the design of effective compression methods with side information is based on generalisations of the Lempel-Ziv family of algorithms [9,10,11,12,13]. A different approach based on grammar-based codes was developed in [14], turbo codes were applied in [15], and a generalised version of context-tree weighting was used in [16].

In this work, we examine the theoretical fundamental limits of the best possible performance that can be achieved in such problems. Let $\left(X,Y\right)=\{\left(X_{n},Y_{n}\right)\;;\;n\geq 1\}$ be a source-side information pair; ***X*** is the source to be compressed, and ***Y*** is the associated side information process which is assumed to be available both to the encoder and the decoder. Under appropriate conditions, the best *average* rate that can be achieved asymptotically [2], is the *conditional entropy rate*,

$$H\left(X|Y\right)=\lim_{n\to \infty }\frac{1}{n}H\left(X_{1}^{n}|Y_{1}^{n}\right),\;\;\mathrm{bits}/\mathrm{symbol},$$

where $X_{1}^{n}=\left(X_{1},X_{2},\ldots ,X_{n}\right)$, $Y_{1}^{n}=\left(Y_{1},Y_{2},\ldots ,Y_{n}\right)$, and $H(X_{1}^{n}|Y_{1}^{n})$ denotes the conditional entropy of $X_{1}^{n}$ given $Y_{1}^{n}$; precise definitions will be given in Section 2.

Our main goal is to derive sharp asymptotic expressions for the optimum compression rate (with side information available to both the encoder and decoder), not only in expectation but with probability 1. In addition to the best first-order performance, we also determine the best rate at which this performance can be achieved, as a function of the length of the data being compressed. Furthermore, we consider an idealised version of a Lempel-Ziv compression algorithm, and we show that it can achieve asymptotically optimal first- and second-order performance, *universally* over a broad class of stationary and ergodic source-side information pairs (X,Y).

Specifically, we establish the following. In Section 2.1 we describe the theoretically optimal one-to-one compressor $f_{n}^{*}\left(X_{1}^{n}|Y_{1}^{n}\right)$, for arbitrary source-side information pairs (X,Y). In Section 2.2 we prove our first result, stating that the description lengths $\ell (f_{n}^{*}\left(X_{1}^{n}|Y_{1}^{n}\right))$ can be well-approximated, with probability one, by the *conditional information density*, $-\log P(X_{1}^{n}|Y_{1}^{n})$. Theorem 2 states that for any jointly stationary and ergodic source-side information pair (X,Y), the best asymptotically achievable compression rate is H(X|Y) bits/symbol, with probability 1. This generalises Kieffer’s corresponding result [17] to the case of compression with side information.

Furthermore, in Section 2.4 we show that there is a sequence of random variables $\left\{Z_{n}\right\}$ such that the description lengths $\ell (f_{n}\left(X_{1}^{n}|Y_{1}^{n}\right))$ of *any* sequence of compressors $\left\{f_{n}\right\}$ satisfy a “one-sided” central limit theorem (CLT): Eventually, with probability 1,

(1) $$\begin{array}{c} \ell \left(f_{n}\left(X_{1}^{n}|Y_{1}^{n}\right)\right)\geq nH\left(X|Y\right)+\sqrt{n}Z_{n}+o\left(\sqrt{n}\right),\;\mathrm{bits}, \end{array}$$

where the $Z_{n}$ converge to a $N(0,\sigma^{2}\left(X|Y\right))$ distribution, and the term $o(\sqrt{n})$ is negligible compared to $\sqrt{n}$. The lower bound (1) is established in Theorem 3 where it is also shown that it is asymptotically achievable. This means that the rate obtained by any sequence of compressors has inevitable $O(\sqrt{n})$ fluctuations around the conditional entropy rate, and that the size of these fluctuations is quantified by the *conditional varentropy rate*,

$$\sigma^{2}\left(X|Y\right)=\lim_{n\to \infty }\frac{1}{n}\mathrm{Var}\left(-\log P(X_{1}^{n}|Y_{1}^{n})\right).$$

This generalises the *minimal coding variance* of [18]. The bound (1) holds for a broad class of source-side information pairs, including all Markov chains with positive transition probabilities. Under the same conditions, a corresponding “one-sided” law of the iterated logarithm (LIL) is established in Theorem 4, which gives a precise description of the inevitable almost-sure fluctuations above H(X|Y), for any sequence of compressors.

The proofs of all the results in Section 2.3 and Section 2.4 are based, in part, on analogous asymptotics for the conditional information density, $-\log P(X_{1}^{n}|Y_{1}^{n})$. These are established in Section 2.5, where we state and prove a corresponding CLT and an LIL for $-\log P(X_{1}^{n}|Y_{1}^{n})$. These results, in turn, follow from the almost sure invariance principle for $-\log P(X_{1}^{n}|Y_{1}^{n})$, proved in Appendix A. Theorem A1, which is of independent interest, generalises the invariance principle established for the (unconditional) information density $-\log P(X_{1}^{n})$ by Philipp and Stout [19]. In fact, Theorem A1 along with the identification of the conditions under which it holds (Assumption 1) in Section 2.4), are the more novel contributions of this work.

In a different direction, Nomura and Han [20] establish finer coding theorems for the Slepian-Wolf problem, when the side information is only available to the decoder. There, they obtain general second-order asymptotics for the best achievable rate region, under an excess-rate probability constraint.

Section 3 is devoted to *universal* compression. We consider a simple, idealised version of Lempel-Ziv coding with side information. As in the case of Lempel-Ziv compression without side information [21,22], the performance of this scheme is determined by the asymptotics of a family of *conditional recurrence times*, $R_{n}=R_{n}\left(X|Y\right)$. Under appropriate, general conditions on the source-side information pair (X,Y), in Theorem 8 we show that the ideal description lengths, $\log R_{n}$, can be well-approximated by the conditional information density $-\log P(X_{1}^{n}|Y_{1}^{n})$. Combining this with our earlier results on the conditional information density, in Corollary 1 and Theorem 9 we show that the compression rate of this scheme converges to H(X|Y), with probability 1, and that it is universally second-order optimal. The results of this section generalise the corresponding asymptotics without side information established in [23,24].

The proofs of the more technical results needed in Section 2 and Section 3 are given in the appendix.

## 2. Pointwise Asymptotics

In this section, we derive general, fine asymptotic bounds for the description lengths of arbitrary compressors with side information, as well as corresponding achievability results.

### 2.1. Preliminaries

Let $X=\{X_{n}\;;\;n\geq 1\}$ be an arbitrary source to be compressed, and $Y=\{Y_{n}\;;\;n\geq 1\}$ be an associated side information process. We let X,Y, denote their finite alphabets, respectively, and we refer to the joint process $\left(X,Y\right)=\{\left(X_{n},Y_{n}\right)\;;\;n\geq 1\}$ as a *source-side information pair*.

Let $x_{1}^{n}=\left(x_{1},x_{2},\ldots ,x_{n}\right)$ be a source string, and let $y_{1}^{n}=\left(y_{1},y_{2},\ldots ,y_{n}\right)$ an associated side information string which is available to both the encoder and decoder. A *fixed-to-variable one-to-one compressor with side information*, of blocklength *n*, is a collection of functions $f_{n}$, where each $f_{n}\left(x_{1}^{n}|y_{1}^{n}\right)$ takes a value in the set of all finite-length binary strings,

$${\left\{0,1\right\}}^{*}=\overset{\infty }{\underset{k=0}{U}}{\left\{0,1\right\}}^{k}=\left\{Ø,0,1,00,01,000,\ldots \right\},$$

with the convention that ${\left\{0,1\right\}}^{0}=\left\{Ø\right\}$ consists of just the empty string Ø of length zero. For each $y_{1}^{n}\in Y^{n}$, we assume that $f_{n}\left(\cdot |y_{1}^{n}\right)$ is a one-to-one function from $X^{n}$ to ${\left\{0,1\right\}}^{*}$, so that the compressed binary string $f_{n}\left(x_{1}^{n}|y_{1}^{n}\right)$ is always correctly decodable.

The main figure of merit in lossless compression is of course the description length,

$$\ell \left(f_{n}\left(x_{1}^{n}|y_{1}^{n}\right)\right)=\mathrm{length}\;\mathrm{of}\;f_{n}\left(x_{1}^{n}|y_{1}^{n}\right),\;\mathrm{bits},$$

where throughout, ℓ(s) denotes the length, in bits, of a binary string *s*. It is easy to see that under quite general criteria, the optimal compressor $f_{n}^{*}$ is easy to describe; see [25] for an extensive discussion. For 1≤i≤j≤∞, we use the shorthand notation $z_{i}^{j}$ for the string $\left(z_{i},z_{i+1},\ldots ,z_{j}\right)$, and similarly $Z_{i}^{j}$ for the corresponding collection of random variables $Z_{i}^{j}=\left(Z_{i},Z_{i+1},\ldots ,Z_{j}\right)$.

**Definition** **1** (The optimal compressor $f_{n}^{*}$). *For each side information string $y_{1}^{n}$, $f_{n}^{*}\left(\cdot |y_{1}^{n}\right)$ is the optimal compressor for the distribution $P(X_{1}^{n}=\cdot |Y_{1}^{n}=y_{1}^{n})$, namely the compressor that orders the strings $x_{1}^{n}$ in order of decreasing probability $P(X_{1}^{n}=x_{1}^{n}|Y_{1}^{n}=y_{1}^{n})$, and assigns them codewords from ${\left\{0,1\right\}}^{*}$ in lexicographic order.*

### 2.2. The Conditional Information Density

**Definition** **2** (Conditional information density). *For an arbitrary source-side information pair (X,Y), the* conditional information density *of blocklength n is the random variable: $-\log P\left(X_{1}^{n}|Y_{1}^{n}\right)=-\log P_{X_{1}^{n}|Y_{1}^{n}}\left(X_{1}^{n}|Y_{1}^{n}\right)$.*

[Throughout the paper, ‘log’ denotes ‘$\log_{2}$’, the logarithm taken to base 2, and all familiar information theoretic quantities are expressed in bits.]

The starting point is the following almost sure (a.s.) approximation result between the description lengths $\ell (f_{n}\left(X_{1}^{n}|Y_{1}^{n}\right))$ of an arbitrary sequence of compressors and the conditional information density $-\log P(X_{1}^{n}|Y_{1}^{n}))$ of an arbitrary source-side information pair (X,Y). When it causes no confusion, we drop the subscripts for PMFs and conditional PMFs, e.g., simply writing $P(x_{1}^{n}|y_{1}^{n})$ for $P_{X_{1}^{n}|Y_{1}^{n}}\left(x_{1}^{n}|y_{1}^{n}\right)$ as in the definition above. Recall the definition of the optimal compressors $\left\{f_{n}^{*}\right\}$ from Section 2.1.

**Theorem** **1.** *For any source-side information pair (X,Y), and any sequence $\left\{B_{n}\right\}$ that grows faster than logarithmically, i.e., such that $B_{n}/\log n\to \infty$ as n→∞, we have:*

(*a*)  *For any sequence of compressors with side information $\left\{f_{n}\right\}$:*
  $$\begin{array}{c} \underset{n\to \infty }{\lim\;\inf}\frac{\ell \left(f_{n}\left(X_{1}^{n}|Y_{1}^{n}\right)\right)-\left[-\log P(X_{1}^{n}|Y_{1}^{n})\right]}{B_{n}}\geq 0,\;a.s. \end{array}$$
(*b*)  *The optimal compressors $\left\{f_{n}^{*}\right\}$ achieve the above bound with equality.*

**Proof.**  Fix ϵ>0 arbitrary and let $\tau =\tau_{n}=\epsilon B_{n}$. Applying the general converse in ([25], Theorem 3.3) with $X_{1}^{n},Y_{1}^{n}$ in place of X,Y and $X^{n},Y^{n}$ in place of X,Y, gives,

$$P\left[\ell \left(f\left(X_{1}^{n}|Y_{1}^{n}\right)\right)\leq -\log P(X_{1}^{n}|Y_{1}^{n})-\epsilon B_{n}\right]\leq 2^{\log n-\epsilon B_{n}}\left(\left\lfloor \log\left|X\right|\right\rfloor +1\right),$$

which is summable in *n*. Therefore, by the Borel-Cantelli lemma we have that eventually, a.s.,

$$\ell \left(f\left(X_{1}^{n}|Y_{1}^{n}\right)\right)+\log P(X_{1}^{n}|Y_{1}^{n})>-\epsilon B_{n},$$

Since ϵ>0 was arbitrary, this implies (a). Part (b) follows from (a) together with the fact that $\ell \left(f_{n}^{*}\left(X_{1}^{n}|Y_{1}^{n}\right)\right)+\log P(X_{1}^{n}|Y_{1}^{n})\leq 0$, a.s., by the general achievability result in ([25], Theorem 3.1). □

### 2.3. First-Order Asymptotics

For any source-side information pair (X,Y), the *conditional entropy rate* H(X|Y) is defined as:

$$H\left(X|Y\right)=\underset{n\to \infty }{\lim\;\sup}\frac{1}{n}H\left(X_{1}^{n}|Y_{1}^{n}\right).$$

Throughout H(Z) and H(Z|W) denote the discrete entropy of *Z* and the conditional entropy of *Z* given *W*, in bits. If (X,Y) are jointly stationary, then the above limsup is in fact a limit, and it is equal to H(X,Y)−H(Y), where H(X,Y) and H(Y) are the entropy rates of (X,Y) and of ***Y***, respectively [2]. Moreover, if (X,Y) are also jointly ergodic, then by applying the Shannon-McMillan-Breiman theorem [2] to ***Y*** and to the pair (X,Y), we obtain its conditional version:

(2) $$-\frac{1}{n}\log P(X_{1}^{n}|Y_{1}^{n})\to H\left(X|Y\right),\;a.s.$$

The next result states that the conditional entropy rate is the best asymptotically achievable compression rate, not only in expectation but also with probability 1. It is a consequence of Theorem 1 with $B_{n}=n$, combined with (2).

**Theorem** **2.** *Suppose (X,Y) is a jointly stationary and ergodic source-side information pair with conditional entropy rate H(X|Y).*

(*a*)  *For any sequence of compressors with side information $\left\{f_{n}\right\}$:*
  $$\begin{array}{c} \underset{n\to \infty }{\lim\;\inf}\frac{\ell (f_{n}\left(X_{1}^{n}|Y_{1}^{n}\right))}{n}\geq H\left(X|Y\right),\;a.s. \end{array}$$
(*b*)  *The optimal compressors $\left\{f_{n}^{*}\right\}$ achieve the above bound with equality.*

### 2.4. Finer Asymptotics

The refinements of Theorem 2 presented in this section will be derived as consequences of the general approximation results in Theorem 1, combined with corresponding refined asymptotics for the conditional information density $-\log P(X_{1}^{n}|Y_{1}^{n})$. For clarity of exposition these are stated separately, in Section 2.5 below.

The results of this section will be established for a class of jointly stationary and ergodic source-side information pairs (X,Y), that includes all Markov chains with positive transition probabilities. The relevant conditions, in their most general form, will be given in terms of the following mixing coefficients.

**Definition** **3.** *Suppose $Z=\{Z_{n}\;;\;n\in Z\}$ is a stationary process on a finite alphabet Z. For any pair of indices −∞≤i≤j≤∞, let $F_{i}^{j}$ denote the σ-algebra generated by $Z_{i}^{j}$. For d≥1, define:*

$$\begin{array}{cc} \alpha^{\left(Z\right)}\left(d\right) & =\sup\left\{|P\left(A\cap B\right)-P\left(A\right)P\left(B\right)|\;;\;A\in F_{-\infty }^{0},B\in F_{d}^{\infty }\right\},\\ \gamma^{\left(Z\right)}\left(d\right) & =\max_{z\in Z}E\left(|\log P(Z_{0}=z|Z_{-\infty }^{-1})-\log P(Z_{0}=z|Z_{-d}^{-1})|\right). \end{array}$$

Note that if ***Z*** is an ergodic Markov chain of order *k*, then $\alpha^{\left(Z\right)}\left(d\right)$ decays exponentially fast [26], and $\gamma^{\left(Z\right)}\left(d\right)=0$ for all d≥k. Moreover, if (X,Y) is a Markov chain with all positive transition probabilities, then $\gamma^{\left(Y\right)}\left(d\right)$ also decays exponentially fast; cf. ([27], Lemma 2.1).

Throughout this section we will assume that the following conditions hold:

**Assumption** **1.** *The source-side information pair (X,Y) is stationary and satisfies one of the following three conditions:*

(a) *(X,Y) is a Markov chain with all positive transition probabilities; or*
(b) *(X,Y) as well as ***Y*** are kth order, irreducible and aperiodic Markov chains; or*
(c) *(X,Y) is jointly ergodic and satisfies the following mixing conditions: [Our source-side information pairs (X,Y) are only defined for $\left(X_{n},Y_{n}\right)$ with n≥1, whereas the coefficients $\alpha^{\left(Z\right)}\left(d\right)$ and $\gamma^{\left(Z\right)}\left(d\right)$ are defined for two-sided sequences $\left\{Z_{n}\;;\;n\in Z\right\}$. However, this does not impose an additional restriction, since any one-sided stationary process can be extended to a two-sided one by the Kolmogorov extension theorem [28].]*
  (3) $$\begin{array}{c} \alpha^{\left(X,Y\right)}\left(d\right)=O\left(d^{-336}\right),\;\gamma^{\left(X,Y\right)}\left(d\right)=O\left(d^{-48}\right),\;and\;\gamma^{\left(Y\right)}\left(d\right)=O\left(d^{-48}\right). \end{array}$$

In view of the discussion following Definition 3, (a)⇒(c) and (b)⇒(c). Therefore, all results stated under Assumption 1 will be proved under the weakest set of conditions, namely that (3) hold.

**Definition** **4.** *For a source-side information pair (X,Y), the* conditional varentropy rate *is:*

(4) $$\sigma^{2}\left(X|Y\right)=\underset{n\to \infty }{\lim\;\sup}\frac{1}{n}\mathrm{Var}\left(-\log P(X_{1}^{n}|Y_{1}^{n})\right).$$

Under the above assumptions, the limsup in (4) is in fact a limit. Lemma 1 is proved in the Appendix A.

**Lemma** **1.** *Under Assumption 1, the conditional varentropy rate $\sigma^{2}\left(X|Y\right)$ is:*

$$\sigma^{2}\left(X|Y\right)=\lim_{n\to \infty }\frac{1}{n}\mathrm{Var}\left(-\log P(X_{1}^{n}|Y_{1}^{n})\right)=\lim_{n\to \infty }\frac{1}{n}\mathrm{Var}\left(-\log\left(\frac{P(X_{1}^{n},Y_{1}^{n}|X_{-\infty }^{0},Y_{-\infty }^{0})}{P(Y_{1}^{n}|Y_{-\infty }^{0})}\right)\right).$$

Our first main result in this section is a “one-sided” central limit theorem (CLT), which states that the description lengths $\ell (f_{n}\left(X_{1}^{n}|Y_{1}^{n}\right))$ of an arbitrary sequence of compressors with side information, $\left\{f_{n}\right\}$, are asymptotically at best Gaussian, with variance $\sigma^{2}\left(X|Y\right)$. Recall the optimal compressors $\left\{f_{n}^{*}\right\}$ described in Section 2.1

**Theorem** **3** (CLT for codelengths). *Suppose (X,Y) satisfy Assumption 1, and let $\sigma^{2}=\sigma^{2}\left(X|Y\right)>0$ denote the conditional varentropy rate* (4)*. Then there exists a sequence of random variables $\left\{Z_{n}\;;\;n\geq 1\right\}$ such that:*

(*a*)  *For any sequence of compressors with side information, $\left\{f_{n}\right\}$, we have,*
  (5) $$\underset{n\to \infty }{\lim\;\inf}\left[\frac{\ell \left(f_{n}\left(X_{1}^{n}|Y_{1}^{n}\right)\right)-H\left(X_{1}^{n}|Y_{1}^{n}\right)}{\sqrt{n}}-Z_{n}\right]\geq 0,\;a.s.,$$
  where $Z_{n}\to N\left(0,\sigma^{2}\right),$ in distribution, as n→∞.
(*b*)  *The optimal compressors $\left\{f_{n}^{*}\right\}$ achieve the lower bound in* (5) *with equality.*

**Proof.**  Letting $Z_{n}=\left[-\log P\left(X_{1}^{n}|Y_{1}^{n}\right)\right]/\sqrt{n}$, n≥1, and taking $B_{n}=\sqrt{n}$, both results follow by combining the approximation results of Theorem 1 with the corresponding CLT for the conditional information density in Theorem 5. □

Our next result is in the form of a “one-sided” law of the iterated logarithm (LIL) which states that with probability 1, the description lengths of any compressor with side information will have inevitable fluctuations of order $\sqrt{2\sigma^{2}n\log_{e}\log_{2}n}$ bits around the conditional entropy rate H(X|Y); throughout, $\log_{e}$ denotes the natural logarithm to base *e*.

**Theorem** **4** (LIL for codelengths). *Suppose (X,Y) satisfy Assumption 1, and let $\sigma^{2}=\sigma^{2}\left(X|Y\right)>0$ denote the conditional varentropy rate* (4)*. Then:*

(*a*)  *For any sequence of compressors with side information, $\left\{f_{n}\right\}$, we have:*
  (6) $$\begin{array}{cc} \; & \underset{n\to \infty }{\lim\;\sup}\frac{\ell \left(f_{n}\left(X_{1}^{n}|Y_{1}^{n}\right)\right)-H\left(X_{1}^{n}|Y_{1}^{n}\right)}{\sqrt{2n\log_{e}\log_{e}n}}\geq \sigma ,\;a.s., \end{array}$$
  (7) $$\begin{array}{cc} and\;\; & \underset{n\to \infty }{\lim\;\inf}\frac{\ell \left(f_{n}\left(X_{1}^{n}|Y_{1}^{n}\right)\right)-H\left(X_{1}^{n}|Y_{1}^{n}\right)}{\sqrt{2n\log_{e}\log_{e}n}}\geq -\sigma ,\;a.s. \end{array}$$
(*b*)  *The optimal compressors $\left\{f_{n}^{*}\right\}$ achieve the lower bounds in* (6) *and* (7) *with equality.*

**Proof.**  Taking $B_{n}=\sqrt{2n\log_{2}\log_{e}n}$, the results of the theorem again follow by combining the approximation results of Theorem 1 with the corresponding LIL for the conditional information density in Theorem 6. □

**Remark** **1.**

1. *Although the results in Theorems 3 and 4 are stated for one-to-one compressors $\left\{f_{n}\right\}$, they remain valid for the class of prefix-free compressors. Since prefix-free codes are certainly one-to-one, the converse bounds in Theorem 3 (a) and 4 (a) are valid as stated, while for the achievability results it suffices to consider compressors $f_{n}^{p}$ with description lengths $\ell \left(f_{n}^{p}\left(x_{1}^{n}|y_{1}^{n}\right)\right))=\left\lceil -\log P\left(x_{1}^{n}|y_{1}^{n}\right)\right\rceil$, and then apply Theorem 5.*
2. *Theorem 3 says that the compression rate of any sequence of compressors $\left\{f_{n}\right\}$ will have at best Gaussian fluctuations around H(X|Y),*
  $$\frac{1}{n}\ell \left(f_{n}^{*}\left(X_{1}^{n}|Y_{1}^{n}\right)\right)\approx N\left(H\left(X|Y\right),\frac{\sigma^{2}\left(X|Y\right)}{n}\right),\;bits/symbol,$$
*and similarly Theorem 4 says that with probability 1, the description lengths will have inevitable fluctuations of approximately $\pm \sqrt{2n\sigma^{2}\log_{e}\log_{e}n}$ bits around nH(X|Y).*
*As both of these vanish when $\sigma^{2}\left(X|Y\right)$ is zero, we note that if the source-side information pair (X,Y) is memoryless, so that $\left\{\left(X_{n},Y_{n}\right)\right\}$ are independent and identically distributed, then the conditional varentropy rate reduces to,*
  $$\sigma^{2}\left(X|Y\right)=\mathrm{Var}\left(-\log P\left(X_{1}|Y_{1}\right)\right),$$
*which is equal to zero if and only if, for each y∈Y, the conditional distribution of $X_{1}$ given $Y_{1}=y$ is uniform on a subset $X_{y}\subset X$, where all the $X_{y}$ have the same cardinality.*
*In the more general case when both the pair process (X,Y) and the side information ***Y*** are Markov chains, necessary and sufficient conditions for $\sigma^{2}\left(X|Y\right)$ to be zero were recently established in [25].*
3. *In analogy with the source dispersion for the problem of lossless compression without side information [29,30], for an arbitrary source-side information pair (X,Y) the conditional dispersion D(X|Y) was recently defined [25] as,*
  $$D\left(X|Y\right)=\underset{n\to \infty }{\lim\;\sup}\frac{1}{n}\mathrm{Var}[\ell (f_{n}^{*}\left(X_{1}^{n}|Y_{1}^{n}\right))].$$
*There, it was shown that when both the pair (X,Y) and **Y** itself are irreducible and aperiodic Markov chains, the conditional dispersion coincides with the conditional varentropy rate:*
  $$D\left(X|Y\right)\;=\lim_{n\to \infty }\frac{1}{n}\mathrm{Var}[\ell (f_{n}^{*}\left(X_{1}^{n}|Y_{1}^{n}\right))]=\sigma^{2}\left(X|Y\right)<\infty .$$

### 2.5. Asymptotics of the Conditional Information Density

Here we show that the conditional information density itself, $-\log P(X_{1}^{n}|Y_{1}^{n})$, satisfies a CLT and a LIL. The next two theorems are consequences of the almost sure invariance principle established in Theorem A1, in the Appendix A.

**Theorem** **5** (CLT for the conditional information density). *Suppose (X,Y) satisfy Assumption 1, and let $\sigma^{2}=\sigma^{2}\left(X|Y\right)>0$ denote the conditional varentropy rate* (4)*. Then, as n→∞:*

(8) $$\frac{-\log P(X_{1}^{n}|Y_{1}^{n})-H\left(X_{1}^{n}|Y_{1}^{n}\right)}{\sqrt{n}}\to N\left(0,\sigma^{2}\right),\;in\;distribution.$$

**Proof.**  The conditions (3), imply that as n→∞, $\left[nH\left(X,Y\right)-H\left(X_{1}^{n},Y_{1}^{n}\right)\right]/\sqrt{n}\to 0$, and $\left[nH\left(Y\right)-H\left(Y_{1}^{n}\right)\right]/\sqrt{n}\to 0$, cf. [19], therefore also, $\left[nH\left(X|Y\right)-H\left(X_{1}^{n}|Y_{1}^{n}\right)\right]/\sqrt{n}\to 0$, so it suffices to show that as n→∞,

(9) $$\frac{-\log P(X_{1}^{n}|Y_{1}^{n})-nH\left(X|Y\right)}{\sqrt{n}}\to N\left(0,\sigma^{2}\right).\;\mathrm{in}\;\mathrm{distribution}.$$

Let D=D([0,1],R) denote the space of *cadlag* (right-continuous with left-hand limits) functions from [0,1] to R, and define, for each t≥0, $S\left(t\right)=\log P(X_{1}^{\left\lfloor t\right\rfloor }|Y_{1}^{\left\lfloor t\right\rfloor })+tH\left(X|Y\right)$, as in Theorem A1 in the Appendix A. For all n≥1,t∈[0,1], define $S_{n}\left(t\right)=S\left(nt\right)$. Then Theorem A1 implies that as n→∞,

$$\left\{\frac{1}{\sigma \sqrt{n}}S_{n}\left(t\right)\;;\;t\in \left[0,1\right]\right\}\to \left\{B\left(t\right)\;;\;t\in \left[0,1\right]\right\},\;\;\mathrm{weakly}\;\mathrm{in}\;D,$$

where {B(t)} is a standard Brownian motion; see, e.g., ([19], Theorem E, p. 4). In particular, this implies that

$$\frac{1}{\sigma \sqrt{n}}S_{n}\left(1\right)\to B\left(1\right)∼N\left(0,1\right),\;\mathrm{in}\;\mathrm{distribution},$$

which is exactly (9). □

**Theorem** **6** (LIL for the conditional information density). *Suppose (X,Y) satisfy Assumption 1, and let $\sigma^{2}=\sigma^{2}\left(X|Y\right)>0$ denote the conditional varentropy rate* (4)*. Then:*

(10) $$\begin{array}{cc} \; & \underset{n\to \infty }{\lim\;\sup}\frac{-\log P(X_{1}^{n}|Y_{1}^{n})-H\left(X_{1}^{n}|Y_{1}^{n}\right)}{\sqrt{2n\log_{e}\log_{e}n}}=\sigma ,\;a.s., \end{array}$$

(11) $$\begin{array}{cc} and\;\; & \underset{n\to \infty }{\lim\;\inf}\frac{-\log P(X_{1}^{n}|Y_{1}^{n})-H\left(X_{1}^{n}|Y_{1}^{n}\right)}{\sqrt{2n\log_{e}\log_{e}n}}=-\sigma ,\;a.s. \end{array}$$

**Proof.**  As in the proof of (8), it suffices to prove (10) with nH(X|Y) in place of $H(X_{1}^{n}|Y_{1}^{n})$. However, this is immediate from Theorem A1, since, for a standard Brownian motion {B(t)},

$$\underset{t\to \infty }{\lim\;\sup}\frac{B(t)}{\sqrt{2t\log_{e}\log_{e}t}}=1,\;a.s.,$$

see, e.g., ([31], Theorem 11.18). In addition, similarly for (11). □

## 3. Idealised LZ Compression with Side Information

Consider the following idealised version of Lempel-Ziv-like compression with side information. For a given source-side information pair $\left(X,Y\right)=\{\left(X_{n},Y_{n}\right)\;;\;n\in Z\}$, the encoder and decoder both have access to the infinite past $\left(X_{-\infty }^{0},Y_{-\infty }^{0}\right)$ and to the current side information $Y_{1}^{n}$. The encoder describes $X_{1}^{n}$ to the decoder as follows. First she searches for the first appearance of $\left(X_{1}^{n},Y_{1}^{n}\right)$ in the past $\left(X_{-\infty }^{0},Y_{-\infty }^{0}\right)$, that is, for the first r≥1 such that $\left(X_{-r+1}^{-r+n},Y_{-r+1}^{-r+n}\right)=\left(X_{1}^{n},Y_{1}^{n}\right)$. Then she counts how many times $Y_{1}^{n}$ appears in $Y_{-\infty }^{0}$ between locations −r+1 and 0, namely how many indices 1≤j<r there are, such that $Y_{-j+1}^{-j+n}=Y_{1}^{n}$. Say there are $\left(R_{n}-1\right)$ such *j*s. She describes $X_{1}^{n}$ to the decoder by telling him to look at the $R_{n}$th position where $Y_{1}^{n}$ appears in the past $Y_{-\infty }^{0}$, and read off the corresponding *X* string.

This description takes $\approx \log R_{n}$ bits, and, as it turns out, the resulting compression rate is asymptotically optimal: As n→∞, with probability 1,

(12) $$\begin{array}{c} \frac{1}{n}\log R_{n}\to H\left(X|Y\right),\;\mathrm{bits}/\mathrm{symbol}. \end{array}$$

Moreover, it is second-order optimal, in that it achieves equality in the CLT and LIL bounds given in Theorems 3 and 4 of Section 2.

Our purpose in this section is to make these statements precise. We will prove (12) as well as its CLT and LIL refinements, generalising the corresponding results for recurrence times without side information in [24].

The use of recurrence times in understanding the Lempel-Ziv (LZ) family of algorithms was introduced by Willems [21] and Wyner and Ziv [22,32]. In terms of practical methods for compression with side information, Subrahmanya and Berger [9] proposed a side information analog of the sliding window LZ algorithm [33], and Uyematsu and Kuzuoka [10] proposed a side information version of the incremental parsing LZ algorithm [34]. The Subrahmanya-Berger algorithm was shown to be asymptotically optimal in [12,13]. Different types of LZ-like algorithms for compression with side information were also considered in [11].

Throughout this section, we assume (X,Y) is a jointly stationary and ergodic source-side information pair, with values in the finite alphabets X,Y, respectively. We use bold lower-case letters x,y without subscripts to denote infinite realizations $x_{-\infty }^{\infty },y_{-\infty }^{\infty }$ of X,Y, and the corresponding bold capital letters X,Y without subscripts to denote the entire process, $X=X_{-\infty }^{\infty },Y=Y_{-\infty }^{\infty }$.

The main quantities of interest are the recurrence times defined next.

**Definition** **5** (Recurrence times). *For a realization **x** of the process **X**, and n≥1, define the* repeated recurrence times *$R_{n}^{\left(j\right)}\left(x\right)$ of $x_{1}^{n}$, recursively, as:*

$$\begin{array}{cc} R_{n}^{\left(1\right)}\left(x\right) & =\inf\{i\geq 1:x_{-i+1}^{-i+n}=x_{1}^{n}\},\\ R_{n}^{\left(j\right)}\left(x\right) & =\inf\{i>R_{n}^{\left(j-1\right)}\left(x\right):x_{-i+1}^{-i+n}=x_{1}^{n}\},\;j>1. \end{array}$$

*For a realization (x,y) of the pair (X,Y) and n≥1, the* joint recurrence time *$R_{n}\left(x,y\right)$ of $\left(x_{1}^{n},y_{1}^{n}\right)$ is defined as,*

$$R_{n}\left(x,y\right)=\inf\left\{i\geq 1:{\left(x,y\right)}_{-i+1}^{-i+n}={\left(x,y\right)}_{1}^{n}\right\},$$

*and the* conditional recurrence time *$R_{n}\left(x|y\right)$ of $x_{1}^{n}$ among the appearances $y_{1}^{n}$ is:*

$$R_{n}\left(x|y\right)=\inf\left\{i\geq 1:x_{-R_{n}^{\left(i\right)}\left(y\right)+1}^{-R_{n}^{\left(i\right)}\left(y\right)+n}=x_{1}^{n}\right\}.$$

An important tool in the asymptotic analysis of recurrence times is Kac’s Theorem [35]. Its conditional version in Theorem 7 was first established in [12] using Kakutani’s induced transformation [36,37].

**Theorem** **7** (Conditional Kac’s theorem). [12] *Suppose (X,Y) is a jointly stationary and ergodic source-side information pair. For any pair of strings $x_{1}^{n}\in X^{n}$, $y_{1}^{n}\in Y^{n}$:*

$$E\left[R_{n}\left(X|Y\right)|X_{1}^{n}=x_{1}^{n},Y_{1}^{n}=y_{1}^{n}\right]=\frac{1}{P(x_{1}^{n}|y_{1}^{n})}.$$

The following result states that we can asymptotically approximate $\log R_{n}\left(X|Y\right)$ by the conditional information density not just in expectation as in Kac’s theorem, but also with probability 1. Its proof is in Appendix B.

**Theorem** **8.** *Suppose (X,Y) is a jointly stationary and ergodic source-side information pair. For any sequence $\left\{c_{n}\right\}$ of non-negative real numbers such that $\sum_{n}n2^{-c_{n}}<\infty$, we have:*

$$\begin{array}{cc} \left(i\right) & \log R_{n}\left(X|Y\right)-\log\left(\frac{1}{P(X_{1}^{n}|Y_{1}^{n})}\right)\leq c_{n},\;\mathrm{eventually}\;a.s.\\ \left(ii\right) & \log R_{n}\left(X|Y\right)-\log\left(\frac{1}{P(X_{1}^{n}|Y_{1}^{n},Y_{-\infty }^{0},X_{-\infty }^{0})}\right)\geq -c_{n},\;\mathrm{eventually}\;a.s.\\ \left(iii\right) & \log R_{n}\left(X|Y\right)-\log\left(\frac{P(Y_{1}^{n}|Y_{-\infty }^{0})}{P(X_{1}^{n},Y_{1}^{n}|Y_{-\infty }^{0},X_{-\infty }^{0})}\right)\geq -2c_{n},\;\mathrm{eventually}\;a.s. \end{array}$$

Next we state the main consequences of Theorem 8 that we will need. Recall the definition of the coefficients $\gamma^{\left(Z\right)}\left(d\right)$ from Section 2.4. Corollary 1 is proved in Appendix B.

**Corollary** **1.** *Suppose (X,Y) are jointly stationary and ergodic.*

(*a*)  *If, in addition, $\sum_{d}\gamma^{\left(X,Y\right)}\left(d\right)<\infty$ and $\sum_{d}\gamma^{\left(Y\right)}\left(d\right)<\infty$, then for any β>0:*
  $$\log\left[R_{n}\left(X|Y\right)P\left(X_{1}^{n}|Y_{1}^{n}\right)\right]=o\left(n^{\beta }\right),\;a.s.$$
(*b*)  *In the general jointly ergodic case, we have:*
  $$\log\left[R_{n}\left(X|Y\right)P\left(X_{1}^{n}|Y_{1}^{n}\right)\right]=o\left(n\right),\;a.s.$$

From part (b) combined with the Shannon-McMillan-Breiman theorem as in (2), we obtain the result (12) promised in the beginning of this section:

$$\lim_{n\to \infty }\frac{1}{n}\log R_{n}\left(X|Y\right)\to H\left(X|Y\right),\;a.s.$$

This was first established in [12]. However, at this point we have already done the work required to obtain much finer asymptotic results for the conditional recurrence time.

For any pair of infinite realizations (x,y) of (X,Y), let $\left\{R^{\left(x|y\right)}\left(t\right)\;;\;t\geq 0\right\}$ be the continuous-time path, defined as:

$$\begin{array}{ccc} R^{\left(x|y\right)}\left(t\right) & = & 0,\;\mathrm{for}\;t<1,\\ R^{\left(x|y\right)}\left(t\right) & = & \log R_{\left\lfloor t\right\rfloor }\left(x|y\right)-\left\lfloor t\right\rfloor H\left(X|Y\right),\;\mathrm{for}\;t\geq 1. \end{array}$$

The following theorem is a direct consequence of Corollary 1 (a) combined with Theorem A1 in the Appendix A. Recall Assumption 1 from Section 2.4.

**Theorem** **9.** *Suppose (X,Y) satisfy Assumption 1, and let $\sigma^{2}=\sigma^{2}\left(X|Y\right)>0$ denote the conditional varentropy rate. Then $\left\{R^{\left(X|Y\right)}\left(t\right)\right\}$ can be redefined on a richer probability space that contains a standard Brownian motion {B(t);t≥0} such that for any λ<1/294:*

$$R^{\left(X|Y\right)}\left(t\right)-\sigma B\left(t\right)=O\left(t^{1/2-\lambda }\right),\;a.s.$$

Two immediate consequences of Theorem 9 are the following:

**Theorem** **10** (CLT and LIL for the conditional recurrence times). *Suppose (X,Y) satisfy Assumption 1, and let $\sigma^{2}=\sigma^{2}\left(X|Y\right)>0$ denote the conditional varentropy rate. Then:*

$$\begin{array}{cc} \left(a\right) & \frac{\log R_{n}\left(X|Y\right)-H\left(X_{1}^{n}|Y_{1}^{n}\right)}{\sqrt{n}}\to N\left(0,\sigma^{2}\right),\;\mathrm{in}\;\mathrm{distribution},\;as\;n\to \infty .\\ \left(b\right) & \underset{n\to \infty }{\lim\;\sup}\frac{\log R_{n}\left(X|Y\right)-H\left(X_{1}^{n}|Y_{1}^{n}\right)}{\sqrt{2n\log_{e}\log_{e}n}}=\sigma ,\;a.s. \end{array}$$

## Appendix A. Invariance Principle for the Conditional Information Density

This Appendix is devoted to the proof of Theorem A1, which generalises the corresponding almost sure invariance principle of Philipp and Stout ([19], Theorem 9.1) for the (unconditional) information density $-\log P(X_{1}^{n})$.

**Theorem** **A1.** *Suppose (X,Y) is a jointly stationary and ergodic process, satisfying the mixing conditions* (3)*. For t≥0, let,*

(A1) $$S\left(t\right)=\log P(X_{1}^{\left\lfloor t\right\rfloor }|Y_{1}^{\left\lfloor t\right\rfloor })+tH\left(X|Y\right).$$

*Then the following series converges:*

$$\begin{array}{cc} \sigma^{2} & =E{\left[\log P(X_{0},Y_{0}|X_{-\infty }^{-1},Y_{-\infty }^{-1})+H\left(X|Y\right)\right]}^{2}\\ \; & +2\sum_{k=1}^{\infty }E\{\left[\log P(X_{0},Y_{0}|X_{-\infty }^{-1},Y_{-\infty }^{-1})+H\left(X|Y\right)\right]\left[\log P(X_{k},Y_{k}|X_{-\infty }^{k-1},Y_{-\infty }^{k-1})+H\left(X|Y\right)\right]\}. \end{array}$$

*If $\sigma^{2}>0$, then, without changing its distribution, we can redefine the process {S(t);t≥0} on a richer probability space that contains a standard Brownian motion {B(t);t≥0}, such that*

(A2) $$S\left(t\right)-\sigma B\left(t\right)=O\left(t^{\frac{1}{2}-\lambda }\right),\;a.s.,$$

*as t→∞, for each λ<1/294.*

To simplify the notation, we write h=H(X|Y) and define,

(A3) $$f_{j}=\log\left(\frac{P(X_{j},Y_{j}|X_{-\infty }^{j-1},Y_{-\infty }^{j-1})}{P(Y_{j}|Y_{-\infty }^{j-1})}\right),\;j\geq 0,$$

so that for example, the variance $\sigma^{2}$ in the theorem becomes,

(A4) $$\begin{array}{c} \sigma^{2}=E\left[{\left(f_{0}+h\right)}^{2}\right]+2\sum_{k=1}^{\infty }E[\left(f_{0}+h\right)\left(f_{k}+h\right)]. \end{array}$$

**Lemma** **A1.**
*If $\sum_{d}\gamma^{\left(X,Y\right)}\left(d\right)<\infty$ and $\sum_{d}\gamma^{\left(Y\right)}\left(d\right)<\infty$ then, as n→∞:*

$$\sum_{k=1}^{n}f_{k}-\log P(X_{1}^{n}|Y_{1}^{n})=O\left(1\right),\;a.s.$$

**Proof.** Let,

$$g_{j}=\log\left(\frac{P(X_{j},Y_{j}|X_{1}^{j-1},Y_{1}^{j-1})}{P(Y_{j}|Y_{1}^{j-1})}\right),\;j\geq 2,$$

and,

$$g_{1}=\log\left(\frac{P(X_{1},Y_{1})}{P(Y_{1})}\right)=\log P\left(X_{1}|Y_{1}\right).$$

We have, for k≥2,

$$\begin{array}{cc} E|f_{k}-g_{k}|\leq & \;E|\log P\left(X_{k},Y_{k}|X_{-\infty }^{k-1},Y_{-\infty }^{k-1}\right)-\log P\left(X_{k},Y_{k}|X_{1}^{k-1},Y_{1}^{k-1}\right)|\\ \; & +E|\log P\left(Y_{k}|Y_{-\infty }^{k-1}\right)-\log P\left(Y_{k}|Y_{1}^{k-1}\right)|\\ \leq & \;\sum_{x,y}E|\log P\left(X_{k}=x,Y_{k}=y|X_{-\infty }^{k-1},Y_{-\infty }^{k-1}\right)-\log P\left(X_{k}=x,Y_{k}=y|X_{1}^{k-1},Y_{1}^{k-1}\right)|\\ \; & +\sum_{y}E|\log P\left(Y_{k}=y|Y_{-\infty }^{k-1}\right)-\log P\left(Y_{k}=y|Y_{1}^{k-1}\right)|\\ \leq & \;|X||Y|\gamma^{\left(X,Y\right)}\left(k-1\right)+\left|Y\right|\gamma^{\left(Y\right)}\left(k-1\right). \end{array}$$

Therefore, $\sum_{k=1}^{\infty }E|f_{k}-g_{k}|<\infty$, and by the monotone convergence theorem we have,

$$\sum_{k=1}^{\infty }|f_{k}-g_{k}|<\infty ,\;a.s.$$

Hence, as n→∞,

$$\left|\sum_{k=1}^{n}f_{k}-\log P(X_{1}^{n}|Y_{1}^{n})\right|\leq \sum_{k=1}^{n}|f_{k}-g_{k}|=O\left(1\right),\;a.s.,$$

as claimed. □

The following bounds are established in the proof of ([19], Theorem 9.1):

**Lemma** **A2.**
*Suppose $Z=\{Z_{n}\;;\;n\in Z\}$ is a stationary and ergodic process on a finite alphabet, with entropy rate H(Z), and such that $\alpha^{\left(Z\right)}\left(d\right)=O\left(d^{-336}\right)$ and $\gamma^{\left(Z\right)}\left(d\right)=O\left(d^{-48}\right),$ as d→∞.*

*Let $f_{k}^{\left(Z\right)}=\log P(Z_{k}|Z_{-\infty }^{k-1})$, k≥0, and put $\eta_{n}^{\left(Z\right)}=f_{n}^{\left(Z\right)}+H\left(Z\right)$, n≥0. Then:*

1. *For each r>0, $E\left[|f_{0}^{\left(Z\right)}{|}^{r}\right]<\infty .$*
2. *For each r≥2 and ϵ>0,*
  $$E\left[|f_{0}^{\left(Z\right)}-\log P(Z_{0}|Z_{-k}^{-1}){|}^{r}\right]\leq C\left(r,\epsilon \right){\left(\gamma^{\left(Z\right)}\left(k\right)\right)}^{\frac{1}{2}-\epsilon },$$
  *where C(r,ϵ) is a constant depending only on r and ϵ.*
3. *For a constant C>0 independent of n, $∥\eta_{n}^{\left(Z\right)}{∥}_{4}\leq C.$*
4. *Let $\eta_{n\ell }^{\left(Z\right)}=E\left[\eta_{n}^{\left(Z\right)}|F_{n-\ell }^{n}\right]$. Then, as ℓ→∞:*
  $$∥\eta_{n}^{\left(Z\right)}-\eta_{n\ell }^{\left(Z\right)}{∥}_{4}=O\left(\ell^{-11/2}\right).$$

Please note that under the assumptions of Theorem A1, the conclusions of Lemma A2 apply to ***Y*** as well as to the pair process (X,Y).

**Lemma** **A3.**
*For each r>0, we have, $E[|f_{0}{|}^{r}]<\infty .$*

**Proof.** Simple algebra shows that

$$f_{0}=f_{0}^{\left(X,Y\right)}-f_{0}^{\left(Y\right)}.$$

Therefore, by two applications of Lemma A2, part 1,

$$∥f_{0}{∥}_{r}\leq ∥f_{0}^{\left(X,Y\right)}{∥}_{r}+{∥f_{0}^{\left(Y\right)}∥}_{r}<\infty .$$

□

The next bound follows from Lemma A2, part 2, upon applying the Minkowski inequality.

**Lemma** **A4.**
*For each r≥2 and each ϵ>0,*

$${∥f_{0}-\log\left(\frac{P(X_{0},Y_{0}|X_{-k}^{-1},Y_{-k}^{-1})}{P(Y_{0}|Y_{-k}^{-1})}\right)∥}_{r}\leq C_{1}\left(r,\epsilon \right){\left[\gamma^{\left(X,Y\right)}\left(k\right)\right]}^{\frac{1-2\epsilon }{2r}}+C_{2}\left(r,\epsilon \right){\left[\gamma^{\left(Y\right)}\left(k\right)\right]}^{\frac{1-2\epsilon }{2r}}.$$

**Lemma** **A5.**
*As N→∞:*

$$E\left\{{\left[\sum_{k\leq N}\left(f_{k}+h\right)\right]}^{2}\right\}=\sigma^{2}N+O\left(1\right).$$

**Proof.** First we examine the definition of the variance $\sigma^{2}$. The first term in (A4),

$$∥f_{0}{+h∥}_{2}^{2}\leq (∥f_{0}{{∥}_{2}+h)}^{2}<\infty ,$$

is finite by Lemma A3. For the series in (A4), let, for k≥0,

$$\phi_{k}=\log\left(\frac{P(X_{k},Y_{k}|X_{\left\lfloor k/2\right\rfloor }^{k-1},Y_{\left\lfloor k/2\right\rfloor }^{k-1})}{P(Y_{k}|Y_{\left\lfloor k/2\right\rfloor }^{k-1})}\right),$$

and write,

(A5) $$\begin{array}{c} E\left(f_{0}+h\right)\left(f_{k}+h\right)=E\left(f_{0}+h\right)\left(f_{k}-\phi_{k}\right)+E\left(f_{0}+h\right)\left(\phi_{k}+h\right). \end{array}$$

For the first term in the right-hand side above, we can bound, for any ϵ>0,

$$\begin{array}{cc} |E\left(f_{0}+h\right)\left(f_{k}-\phi_{k}\right)| & \;\overset{\left(a\right)}{\leq }\;∥f_{0}{+h∥}_{2}{∥f_{k}-\phi_{k}∥}_{2}\\ \; & \leq [∥f_{0}{∥}_{2}+h]∥f_{k}-\phi_{k}{∥}_{2}\\ \; & \;\overset{\left(b\right)}{\leq }\;AC_{1}\left(2,\epsilon \right){\left[\gamma^{\left(X,Y\right)}\left(\left\lfloor k/2\right\rfloor \right)\right]}^{\frac{1}{4}-\frac{1}{2}\epsilon }+AC_{2}\left(2,\epsilon \right){\left[\gamma^{\left(Y\right)}\left(\left\lfloor k/2\right\rfloor \right)\right]}^{\frac{1}{4}-\frac{1}{2}\epsilon }, \end{array}$$

where (a) follows by the Cauchy-Schwarz inequality, and (b) follows by Lemmas A3 and A4, with $A=∥f_{0}{∥}_{2}+h<\infty$. Therefore, taking ϵ>0 small enough and using the assumptions of Theorem A1,

(A6) $$\begin{array}{c} |E\left(f_{0}+h\right)\left(f_{k}-\phi_{k}\right)|=O\left(k^{-12+24\epsilon }\right)=O\left(k^{-3}\right),\;\mathrm{as}\;k\to \infty . \end{array}$$

For the second term in (A5), we have that for any r>0, $∥\phi_{k}{∥}_{r}<\infty$, uniformly over k≥1 by stationarity. Also, since $f_{0},\phi_{k}$ are measurable with respect to the σ-algebras generated by $\left(X_{-\infty }^{0},Y_{-\infty }^{0}\right)$ and $\left(X_{\left\lfloor k/2\right\rfloor }^{\infty },Y_{\left\lfloor k/2\right\rfloor }^{\infty }\right)$, respectively, we can apply ([19], Lemma 7.2.1) with p=r=s=3, to obtain that

$$|E\left(f_{0}+h\right)\left(\phi_{k}+h\right)|\leq 10∥f_{0}{+h∥}_{3}{∥\phi_{k}+h∥}_{3}\alpha {\left(\left\lfloor k/2\right\rfloor \right)}^{1/3},$$

where $\alpha \left(k\right)=\alpha^{\left(X,Y\right)}\left(k\right)=O\left(k^{-48}\right)$, as k→∞, by assumption. Therefore, a fortiori,

$$E\left(f_{0}+h\right)\left(f_{k}+h\right)=O\left(k^{-3}\right),$$

and combining this with (A6) and substituting in (A5), implies that $\sigma^{2}$ in (A4) is well defined and finite. Finally, we have that as N→∞,

$$\begin{array}{cc} E\left\{{\left[\sum_{k\leq N}\left(f_{k}+h\right)\right]}^{2}\right\} & =NE{\left(f_{0}+h\right)}^{2}+2\sum_{k=0}^{N-1}\left(N-k\right)E\left(f_{0}+h\right)\left(f_{k}+h\right)\\ \; & =N\sigma^{2}-2\sum_{k=1}^{N-1}kE\left(f_{0}+h\right)\left(f_{k}+h\right)-2N\sum_{k=N}^{\infty }E\left(f_{0}+h\right)\left(f_{k}+h\right)\\ \; & =\sigma^{2}N+O\left(1\right), \end{array}$$

as required. □

**Proof** **of** **Lemma** **1.** Lemma A5 states that the limit,

(A7) $$\lim_{n\to \infty }\frac{1}{n}\mathrm{Var}\left(-\log\left(\frac{P(X_{1}^{n},Y_{1}^{n}|X_{-\infty }^{0},Y_{-\infty }^{0})}{P(Y_{1}^{n}|Y_{-\infty }^{0})}\right)\right).$$

exists and is finite. Moreover, by Lemma A4, after an application of the Cauchy-Schwarz inequality, we have that as n→∞,

$$E\left\{{\left[\sum_{k\leq n}\left|\log\left(\frac{P(X_{k},Y_{k}|X_{1}^{k-1},Y_{1}^{k-1})}{P(Y_{k}|Y_{1}^{k-1})}\right)-\log\left(\frac{P(X_{k},Y_{k}|X_{-\infty }^{k-1},Y_{-\infty }^{k-1})}{P(Y_{k}|Y_{-\infty }^{k-1})}\right)\right|\right]}^{2}\right\}=O\left(1\right),$$

therefore,

$$\frac{1}{n}\left\{\mathrm{Var}\left(-\log P(X_{1}^{n}|Y_{1}^{n})\right)-\mathrm{Var}\left(-\log\left(\frac{P(X_{1}^{n},Y_{1}^{n}|X_{-\infty }^{0},Y_{-\infty }^{0})}{P(Y_{1}^{n}|Y_{-\infty }^{0})}\right)\right)\right\}=o\left(1\right).$$

Combining this with (A7) and the definition of $\sigma^{2}$, completes the proof. □

**Proof** **of** **Theorem** **A1.** Note that we have already established the fact that the expression for the variance converges to some $\sigma^{2}<\infty$. Also, in view of Lemma A1, it is sufficient to prove the theorem for $\left\{\tilde{S}\left(t\right)\right\}$ instead of {S(t)}, where:

$$\tilde{S}\left(t\right)=\sum_{k\leq t}\left(f_{k}+h\right),\;t\geq 0.$$

This will be established by an application of ([19], Theorem 7.1), once we verify that conditions (7.1.4), (7.1.5), (7.1.6), (7.1.7) and (7.1.9) there are all satisfied. For each n≥0, let $\eta_{n}=f_{n}+h$, where $f_{n}$ is defined in (A3) and *h* is the conditional entropy rate. First we observe that by stationarity,

(A8) $$\begin{array}{cc} E[\eta_{n}] & =E\left[\log\left(\frac{P(X_{n},Y_{n}|X_{-\infty }^{n-1},Y_{-\infty }^{n-1})}{P(Y_{n}|Y_{-\infty }^{n-1})}\right)\right]+H\left(X|Y\right)\\ \; & =E\left[\log P(X_{0},Y_{0}|X_{-\infty }^{-1},Y_{-\infty }^{-1})\right]+H\left(X,Y\right)-E\left[\log P(Y_{0}|Y_{-\infty }^{-1})\right]-H\left(Y\right)\\ \; & =0, \end{array}$$

where H(X,Y) and H(Y) denote the entropy rates of (X,Y) and ***Y***, respectively [2]. Observe that in the notation of Lemma A2, $\eta_{n}=\eta_{n}^{\left(X,Y\right)}-\eta_{n}^{\left(Y\right)}$, and $\eta_{n\ell }=\eta_{n\ell }^{\left(X,Y\right)}-\eta_{n\ell }^{\left(Y\right)}.$ By Lemma A2, parts 3 and 4, there exist a constant *C*, independent of *n* such that

(A9) $$∥\eta_{n}{∥}_{4}\leq C<\infty ,$$

and,

(A10) $$∥\eta_{n}-\eta_{n\ell }{∥}_{4}=O\left(\ell^{-11/2}\right).$$

In addition, from Lemma A5 we have,

(A11) $$E\left\{{\left(\sum_{n\leq N}\frac{1}{\sigma }\eta_{n}\right)}^{2}\right\}=N+O\left(1\right).$$

From (A8)–(A11) and the assumption that $\alpha^{\left(X,Y\right)}\left(d\right)=O\left(d^{-336}\right)$, we have that all of the conditions (7.1.4), (7.1.5), (7.1.6), (7.1.7) and (7.1.9) of ([19], Theorem 7.1) are satisfied for the random variables $\left\{\eta_{n}/\sigma \right\}$, with δ=2. Therefore, $\left\{\tilde{S}\left(t\right)\;;\;t\geq 0\right\}$ can be redefined on a possibly richer probability space, where there exists a standard Brownian motion {B(t);t≥0}, such that as t→∞:

$$\frac{1}{\sigma }\tilde{S}\left(t\right)-B\left(t\right)=O\left(t^{1/2-\lambda }\right),\;a.s.$$

By Lemma A1, this completes the proof. □

## Appendix B. Recurrence Times Proofs

In this appendix, we provide the proofs of some of the more technical results in Section 3. First we establish the following generalisation of ([2], Lemma 16.8.3).

**Lemma** **A6.**
*Suppose (X,Y) is an arbitrary source-side information pair. Then, for any sequence $\left\{t_{n}\right\}$ of non-negative real numbers such that $\sum_{n}2^{-t_{n}}<\infty$, we have:*

$$\log\frac{P(Y_{1}^{n}|Y_{-\infty }^{0},X_{-\infty }^{0})}{P(Y_{1}^{n}|Y_{-\infty }^{0})}\geq -t_{n},\;\mathrm{eventually}\;a.s.$$

**Proof.** Let $B\left(X_{-\infty }^{0},Y_{-\infty }^{0}\right)\subset Y^{n}$ denote the support of $P(\cdot |X_{-\infty }^{0},Y_{-\infty }^{0})$. We can compute,

$$\begin{array}{cc} E\left(\frac{P(Y_{1}^{n}|Y_{-\infty }^{0})}{P(Y_{1}^{n}|Y_{-\infty }^{0},X_{-\infty }^{0})}\right) & =E\left(E\left(\frac{P(Y_{1}^{n}|Y_{-\infty }^{0})}{P(Y_{1}^{n}|Y_{-\infty }^{0},X_{-\infty }^{0})}|Y_{-\infty }^{0},X_{-\infty }^{0}\right)\right)\\ \; & =E\left(\sum_{y_{1}^{n}\in B\left(X_{-\infty }^{0},Y_{-\infty }^{0}\right)}\frac{P(y_{1}^{n}|Y_{-\infty }^{0})}{P(y_{1}^{n}|Y_{-\infty }^{0},X_{-\infty }^{0})}P\left(y_{1}^{n}|Y_{-\infty }^{0},X_{-\infty }^{0}\right)\right)\\ \; & \leq 1. \end{array}$$

By Markov’s inequality,

$$P\left[\log\left(\frac{P(Y_{1}^{n}|Y_{-\infty }^{0})}{P(Y_{1}^{n}|Y_{-\infty }^{0},X_{-\infty }^{0})}\right)>t_{n}\right]=P\left[\frac{P(Y_{1}^{n}|Y_{-\infty }^{0})}{P(Y_{1}^{n}|Y_{-\infty }^{0},X_{-\infty }^{0})}>2^{t_{n}}\right]\leq 2^{-t_{n}},$$

and so, by the Borel-Cantelli lemma,

$$\log\frac{P(Y_{1}^{n}|Y_{-\infty }^{0})}{P(Y_{1}^{n}|Y_{-\infty }^{0},X_{-\infty }^{0})}\leq t_{n},\;\mathrm{eventually}\;a.s.,$$

as claimed. □

**Proof** **of** **Theorem** **8.** Let K>0 arbitrary. By Markov’s inequality and Kac’s theorem,

$$\begin{array}{cc} P(R_{n}\left(X|Y\right)>K|X_{1}^{n}=x_{1}^{n},Y_{1}^{n}=y_{1}^{n}) & \leq \frac{E\left(R_{n}\left(X|Y\right)|X_{1}^{n}=x_{1}^{n},Y_{1}^{n}=y_{1}^{n}\right)}{K}\\ \; & =\frac{1}{KP(x_{1}^{n}|y_{1}^{n})}. \end{array}$$

Taking $K=2^{c_{n}}/P\left(X_{1}^{n}|Y_{1}^{n}\right)$, we obtain,

$$\begin{array}{cc} \; & P\left(\log\left[R_{n}\left(X|Y\right)P\left(X_{1}^{n}|Y_{1}^{n}\right)\right]>c_{n}|X_{1}^{n}=x_{1}^{n},Y_{1}^{n}=y_{1}^{n}\right)\\ \; & =P\left(R_{n}\left(X|Y\right)>\frac{2^{c_{n}}}{P(X_{1}^{n}|Y_{1}^{n})}|X_{1}^{n}=x_{1}^{n},Y_{1}^{n}=y_{1}^{n}\right)\\ \; & \leq 2^{-c_{n}}. \end{array}$$

Averaging over all $x_{1}^{n}\in X^{n},y_{1}^{n}\in Y^{n}$,

$$P(\log R_{n}\left(X|Y\right)P\left(X_{1}^{n}|Y_{1}^{n}\right)>c_{n})\leq 2^{-c_{n}},$$

and the Borel-Cantelli lemma gives (i). For (ii) we first note that the probability,

(A12) $$P\left(\log\left[R_{n}\left(X|Y\right)P\left(X_{1}^{n}|Y_{1}^{n},X_{-\infty }^{0},Y_{-\infty }^{0}\right)\right]<-c_{n}|Y_{1}^{n}=y_{1}^{n},X_{-\infty }^{0}=x_{-\infty }^{0},Y_{-\infty }^{0}=y_{-\infty }^{0}\right)$$

is the probability, under $P(X_{1}^{n}=\cdot |Y_{1}^{n}=y_{1}^{n},X_{-\infty }^{0}=x_{-\infty }^{0},Y_{-\infty }^{0}=y_{-\infty }^{0})$, of those $z_{1}^{n}$ such that

$$\begin{array}{c} P\left(X_{1}^{n}=z_{1}^{n}|X_{-\infty }^{0},Y_{-\infty }^{n}\right)<\frac{2^{-c_{n}}}{R_{n}\left(x_{-\infty }^{0}*z_{1}^{n}|y_{-\infty }^{n}\right)}, \end{array}$$

where ‘*’ denotes the concatenation of strings. Let $G_{n}=G_{n}\left(x_{-\infty }^{0},y_{-\infty }^{n}\right)\subset X^{n}$ denote the set of all such $z_{1}^{n}$. Then the probability in (A12) is,

$$\sum_{z_{n}\in G_{n}}P(z_{1}^{n}|x_{-\infty }^{0},y_{-\infty }^{n})\leq \sum_{z_{n}\in G_{n}}\frac{2^{-c_{n}}}{R_{n}\left(x_{-\infty }^{0}*z_{1}^{n}|y_{-\infty }^{n}\right)}\leq 2^{-c_{n}}\sum_{z_{n}\in X^{n}}\frac{1}{R_{n}\left(x_{-\infty }^{0}*z_{1}^{n}|y_{-\infty }^{n}\right)}.$$

Since both $x_{-\infty }^{0}$ and $y_{-\infty }^{n}$ are fixed, for each j≥1, there is exactly one $z_{1}^{n}\in X^{n}$, such that $R_{n}\left(x_{-\infty }^{0}*z_{1}^{n}|y_{-\infty }^{n}\right)=j$. Thus, the last sum is bound above by,

$$\sum_{j=1}^{{\left|X\right|}^{n}}\frac{1}{j}\leq Dn,$$

for some positive constant *D*. Therefore, the probability in (A12) is bounded above by $Dn2^{-c_{n}}$, which is independent of $x_{-\infty }^{0},y_{-\infty }^{n}$ and, by assumption, summable over *n*. Hence, after averaging over all infinite sequences $x_{-\infty }^{0},y_{-\infty }^{n}$, the Borel-Cantelli lemma gives (ii). For part (iii) we have, eventually, almost surely,

$$\begin{array}{cc} \log & \left[R_{n}\left(X|Y\right)\frac{P(X_{1}^{n},Y_{1}^{n}|Y_{-\infty }^{0},X_{-\infty }^{0})}{P(Y_{1}^{n}|Y_{-\infty }^{0})}\right]\\ \; & =\log\left[R_{n}\left(X|Y\right)\frac{P\left(X_{1}^{n}|Y_{1}^{n},X_{-\infty }^{0},Y_{-\infty }^{0}\right)P\left(Y_{1}^{n}|X_{-\infty }^{0},Y_{-\infty }^{0}\right)}{P(Y_{1}^{n}|Y_{-\infty }^{0})}\right]\\ \; & =\log\left[R_{n}\left(X|Y\right)P\left(X_{1}^{n}|Y_{1}^{n},X_{-\infty }^{0},Y_{-\infty }^{0}\right)\right]+\log\left[\frac{P(Y_{1}^{n}|X_{-\infty }^{0},Y_{-\infty }^{0})}{P(Y_{1}^{n}|Y_{-\infty }^{0})}\right]\;\geq \;-2c_{n}, \end{array}$$

where the last inequality follows from (ii) and Lemma A6, and we have shown (iii). □

**Proof** **of** **Corollary** **1.** If we take $c_{n}=\epsilon n^{\beta }$ in theorem 8, with ϵ>0 arbitrary, we get from (i) and (iii),

(A13) $$\begin{array}{cc} \; & \underset{n\to \infty }{\lim\;\sup}\frac{1}{n^{\beta }}\log[R_{n}\left(X|Y\right)P\left(X_{1}^{n}|Y_{1}^{n}\right)]\leq 0,\;a.s. \end{array}$$

(A14) $$\begin{array}{cc} and\;\; & \underset{n\to \infty }{\lim\;\inf}\frac{1}{n^{\beta }}\log\left[R_{n}\left(X|Y\right)\frac{P(X_{1}^{n},Y_{1}^{n}|X_{-\infty }^{0},Y_{-\infty }^{0})}{P(Y_{1}^{n}|Y_{-\infty }^{0})}\right]\geq 0,\;a.s. \end{array}$$

Hence, to prove (a) it is sufficient to show that as n→∞,

$$\log P(X_{1}^{n}|Y_{1}^{n})-\log\left[\frac{P(X_{1}^{n},Y_{1}^{n}|X_{-\infty }^{0},Y_{-\infty }^{0})}{P(Y_{1}^{n}|Y_{-\infty }^{0})}\right]=O\left(1\right),\;a.s.,$$

which is exactly Lemma A1 in Appendix A. To prove (b), taking β=1 in (A13) and (A14), it suffices to show that

$$\lim_{n\to \infty }\left\{\frac{1}{n}\log P(X_{1}^{n}|Y_{1}^{n})-\frac{1}{n}\log\left(\frac{P(X_{1}^{n},Y_{1}^{n}|X_{-\infty }^{0},Y_{-\infty }^{0})}{P(Y_{1}^{n}|Y_{-\infty }^{0})}\right)\right\}=0,\;a.s.$$

However, the first term converges almost surely to −H(X|Y) by the Shannon-McMillan-Breiman theorem, as in (2), and the second term is,

$$-\frac{1}{n}\sum_{i=1}^{n}\log P(X_{i},Y_{i}|X_{-\infty }^{i-1},Y_{-\infty }^{i-1})+\frac{1}{n}\sum_{i=1}^{n}\log P(Y_{i}|Y_{-\infty }^{i-1}),$$

which, by the ergodic theorem, converges almost surely to,

$$-E\left[\log P(X_{0},Y_{0}|X_{-\infty }^{0},Y_{-\infty }^{0})\right]+E\left[\log P(Y_{0}|Y_{-\infty }^{0})\right]=H\left(X,Y\right)-H\left(Y\right)=H\left(X|Y\right).$$

This completes the proof. □
